# Biodiversity and ecosystem functions depend on environmental conditions and resources rather than the geodiversity of a tropical biodiversity hotspot

**DOI:** 10.1038/s41598-021-03488-1

**Published:** 2021-12-31

**Authors:** Christine I. B. Wallis, Yvonne C. Tiede, Erwin Beck, Katrin Böhning-Gaese, Roland Brandl, David A. Donoso, Carlos I. Espinosa, Andreas Fries, Jürgen Homeier, Diego Inclan, Christoph Leuschner, Mark Maraun, Katrin Mikolajewski, Eike Lena Neuschulz, Stefan Scheu, Matthias Schleuning, Juan P. Suárez, Boris A. Tinoco, Nina Farwig, Jörg Bendix

**Affiliations:** 1 Département de Biologie, Université de Sherbrooke, Sherbrooke, QC J1K 2R1 Canada; 2 Laboratory for Climatology and Remote Sensing, Department of Geography, Philipps-Universität Marburg, Marburg, Germany; 3 Conservation Ecology, Department of Biology, Philipps-Universität Marburg, Marburg, Germany; 4 Hessian Ministry of the Environment, Climate Protection, Agriculture and Consumer Protection, Wiesbaden, Germany; 5 Department of Plant Physiology, Bayreuth Center of Ecology and Environmental Research, University of Bayreuth, Bayreuth, Germany; 6 Senckenberg Biodiversity and Climate Research Centre (SBiK-F), Frankfurt am Main, Germany; 7 Institute for Ecology, Evolution and Diversity, Goethe University, Frankfurt am Main, Germany; 8 Animal Ecology, Department of Biology, Philipps-Universität Marburg, Marburg, Germany; 9 Departamento de Ciencias Biológicas, Escuela Politécnica Nacional, Quito, Ecuador; 10 Centro de Investigación de La Biodiversidad Y Cambio Climático, Universidad Tecnológica Indoamérica, Quito, Ecuador; 11 Laboratorio de Ecología Tropical Y Servicios Ecosistémicos, Universidad Técnica Particular de Loja, Loja, Ecuador; 12 Unidad Ingeniería Civil Y Geología (UCG), Hidrología y Climatología Working Group, Universidad Tecnica Particular de Loja, Loja, Ecuador; 13 Plant Ecology, Albrecht Von Haller Institute for Plant Sciences, University of Göttingen, Göttingen, Germany; 14 Facultad de Ciencias Agrícolas, Universidad Central del Ecuador, Quito, Ecuador; 15 Instituto Nacional de Biodiversidad, Quito, Ecuador; 16 J.F. Blumenbach Institute of Zoology and Anthropology, University of Göttingen, Göttingen, Germany; 17 Atmosfair gGmbH, Berlin, Germany; 18 Centre of Biodiversity and Sustainable Land Use, University of Göttingen, Göttingen, Germany; 19 Departamento de Ciencias Biológicas, Universidad Técnica Particular de Loja, Loja, Ecuador; 20 Escuela de Biología, Universidad del Azuay, Cuenca, Ecuador

**Keywords:** Biodiversity, Environmental sciences, Biodiversity, Conservation biology, Ecological modelling, Forest ecology, Macroecology, Tropical ecology

## Abstract

Biodiversity and ecosystem functions are highly threatened by global change. It has been proposed that geodiversity can be used as an easy-to-measure surrogate of biodiversity to guide conservation management. However, so far, there is mixed evidence to what extent geodiversity can predict biodiversity and ecosystem functions at the regional scale relevant for conservation planning. Here, we analyse how geodiversity computed as a compound index is suited to predict the diversity of four taxa and associated ecosystem functions in a tropical mountain hotspot of biodiversity and compare the results with the predictive power of environmental conditions and resources (climate, habitat, soil). We show that combinations of these environmental variables better explain species diversity and ecosystem functions than a geodiversity index and identified climate variables as more important predictors than habitat and soil variables, although the best predictors differ between taxa and functions. We conclude that a compound geodiversity index cannot be used as a single surrogate predictor for species diversity and ecosystem functions in tropical mountain rain forest ecosystems and is thus little suited to facilitate conservation management at the regional scale. Instead, both the selection and the combination of environmental variables are essential to guide conservation efforts to safeguard biodiversity and ecosystem functions.

## Introduction

Safeguarding biodiversity^1–3^ and ecosystem functions under global change^4,5^ is of key importance for the provision of essential services to human societies^6^. Geodiversity indices (i.e., the diversity of earth system's abiotic and structural features) are increasingly used to predict biodiversity and ecosystem functions^1,2,7^. A comprehensive review on how the term geodiversity evolved and diversified over time is given in ref.^8^. Early concepts of geodiversity proposed the conservation of a broad range of environments to maintain biological diversity^9^, in particular, considering geological features of a region (Supplementary Table S1). These concepts were later extended by a variety of geomorphological, hydrological and pedological features (Supplementary Table S1, see also ref.^10^). To describe geodiversity, simple feature numbers were initially used but later, inspired by biology, replaced by metrics such as the Shannon diversity index^11–13^.

Using geodiversity as a surrogate for biodiversity burgeoned around the new millennium^13^, mainly to identify areas for conservation or natural heritage or to manage the exploitation of ecosystem services^12,14–16^. This endeavour was based on the hypothesis that specific geo-sites should support unique biota so that high geodiversity is coupled with high biodiversity^17–19^. This concept has been particularly addressed by the Conserving Nature's Stage (CNS) approach which focusses on the use of geodiversity in conservation planning^9,18,19^. Because geodiversity was expected to be more resilient against environmental changes than biodiversity, it was claimed that preserving geodiversity should safeguard biodiversity and ecosystem functions and services^20–22^. Consequently, geodiversity data, in parts easily available through remote sensing, were proposed as suitable planning tools to predict biodiversity and ecosystem functions, particularly in the frequent case of biodiversity data unavailability^23–25^.

To date, different types of geodiversity indices are used to predict biodiversity pattern^8^. They range from simple feature-based to complex multivariate indices such as geodiversity indices that consider climate, hydrology, soil, topography and habitat, and their variability in space and time (Supplementary Table S1, see also ref.^23^). Our understanding of the concept of geodiversity is in line with the resource-based measure of geodiversity explained in ref.^23^ (also discussed in ref.^8^). In the following, we will refer to geodiversity as the spatial variation of environmental variables. The environmental variables (also elsewhere termed as abiotic conditions, abiotic variables or abiotic stages^26^) used in this study consider conditions and resources related to climate, habitat and soil which are requirements for the establishment and survival of organisms and have been used as meaningful predictors of species diversity and ecosystem functions (Supplementary Methods). These variables are here used as a direct (the value measured at a plot) or indirect (the diversity of multiple variables at a plot and its surrounding; hereafter the compound geodiversity index) surrogate for species diversity and ecosystem functions (see Methods and Supplementary Methods). Our view on using the environmental variables to calculate a geodiversity compound index is supported by a review on the CNS approach that recommends to consider a broader spectrum of environmental variables at a finer thematic resolution^27^. At the same time, a harmonization of geodiversity approaches has recently been claimed to reach intercomparability of studies focussing on the geodiversity-biodiversity relationship, and further test this relationship for underrepresented ecosystems (such as tropical mountain rain forests)^7^.

While biodiversity is defined by the wealth and variety of life, depending on the scale and the level of complexity, a range of measures can be applied to surrogate it, i.e., the diversity of ecosystems, communities, or taxa^28,29^. So far, most biodiversity studies used measures of species diversity such as species richness, or abundance-weighted diversity metrics, e.g. the Shannon index^30^. This is often due to the simplicity of these indices and the ease with which they can be compared within and across ecosystems. As taxonomic species diversity alone, however, misses much of the overall diversity of an ecosystem^31^, measures of ecosystem functions can be additionally addressed to describe the state of an ecosystem. Ecosystem functions are often defined as the biological, physical, and geochemical processes that occur within an ecosystem and consider the sizes of compartments (such as carbon storage) as well as the rates of their changes (such as rates of carbon sequestration)^32^. They can provide or regulate ecosystem services^32^ and are, therefore, an integral part of the biodiversity of ecosystems^32^.

Using the Shannon index for the calculation of geodiversity and species diversity could thus facilitate the direct comparisons of geodiversity and species diversity. But research on geodiversity-biodiversity relations showed different performance depending on selected ecosystem, taxa, and scale, where geodiversity predictors seemed to generally work better for intermediate to large scales^33–35^. CNS research activities, however, revealed best performance of geo-biodiversity at landscape to regional scales that are most important for conservation planning^26^. Also challenging is often the match of geo- and biodiversity scales. It is important that the environmental variables used for the calculation of a geodiversity compound index are drivers of the species diversity of the taxon under investigation. Since the strength of environmental variables as predictors differs between different taxa, it is unlikely that there is a geodiversity index which can be used as a surrogate of species diversity across multiple taxa. Therefore, the choice of environmental variables to calculate taxon-specific geodiversity indices is of great importance. Not only the environmental variables but also the scales used to calculate the geodiversity compound index should have ecological significance for the taxa and ecosystem functions studied^35^.

Here, we test the hypothesis that geodiversity compound indices are powerful predictors of species diversity and ecosystem functions in a tropical mountain rainforest hotspot for biodiversity. Specifically, we compare predictive models of species diversity and ecosystem functions based on a geodiversity index with those based on environmental variables. In this study we use the following definitions: (i) Environmental variables consider the conditions and resources at each plot. (ii) The geodiversity index is assembled as the sum of the spatial diversity of multiple environmental variables measured within each plot and its surrounding (see Methods and Supplementary Fig. S1). For both, (i) and (ii) environmental variables belong to the three groups climate, habitat, and soil, each comprising a set of different environmental conditions and resources available as spatial grids (Table 1). We consider one condition or resource from each group and subsequently use the selected three environmental variables as predictors of species diversity and ecosystem functions (Extended Data Table E3). For the geodiversity compound index, all spatial grids are classified, and the central pixel of each plot and its adjacent pixels are used to calculate their spatial diversity analogous to the calculation of Shannon diversity (Supplementary Fig. S1). The geodiversity index is subsequently calculated by summing up the diversities of the selected environmental variables indicating the degree of variation across space. Comparison of the geodiversity index models with models of the environmental variables has been conducted for the following taxa: trees, testate amoebae, ants and birds, and their associated ecosystem functions: carbon sequestration, decomposition, predation, and seed dispersal, along an elevational gradient ranging from 1000 to 3000 m a.s.l.

**Table 1:** Potential predictors considering environmental conditions and resources within climate, habitat, and soil variables.

	Environmental variables	Raster source layer/spatial resolution	Calculation/references
**Climate**	Based on averaged monthly air temperature in C°: annual mean (mean), annual maximum (max), annual standard deviation (sd)	Landsat-8 scene, 30 m spatial resolution	After ref.^80^
	Humidity in %: mean annual	Landsat-8 scene, 30 m spatial resolution	After ref.^81^
**Habitat**	Normalized difference vegetation index (NDVI)	Landsat-8 scene, 30 m spatial resolution	(near infrared − red)/(near infrared + red)
	Image textural metric: NDVI correlation	Landsat-8 scene, 30 m spatial resolution	‘glcm’ package in R; using all directions^82,83^
	Red-Blue normalized difference vegetation index (RBVI)	Landsat-8 scene, 30 m spatial resolution	(red − blue)/(red + blue)
	Forest cover in %	Landsat-8 scene, 30 m spatial resolution	Spectral unmixing in IDRISI Andes after^92^
	Topographical position index (TPI)	Sigtierras image, 6 m spatial resolution	After ref.^93^ with a surrounding of 17 pixels
	Leaf area index (LAI)	Sentinel-2 scene, 10 m spatial resolution	S2 SNAP (Sentinel Application Platform) Toolbox Biophysical Processor^94^
**Soil**	pH in Bv-Horizon	30 m spatial resolution	Model prediction; Extended Data Tables E1, E2
	Total phosphorus soil in kg/ha in Ah-Horizon (phosphorus)	30 m spatial resolution	Model prediction; Extended Data Tables E1, E2
	Organic layer depth in cm	30 m spatial resolution	Model prediction; Extended Data Tables E1, E2

Raster source means the underlying raster data that were used to determine the environmental variables in space.

## Results and discussion

### Geodiversity and environmental variables

**Figure 1:**
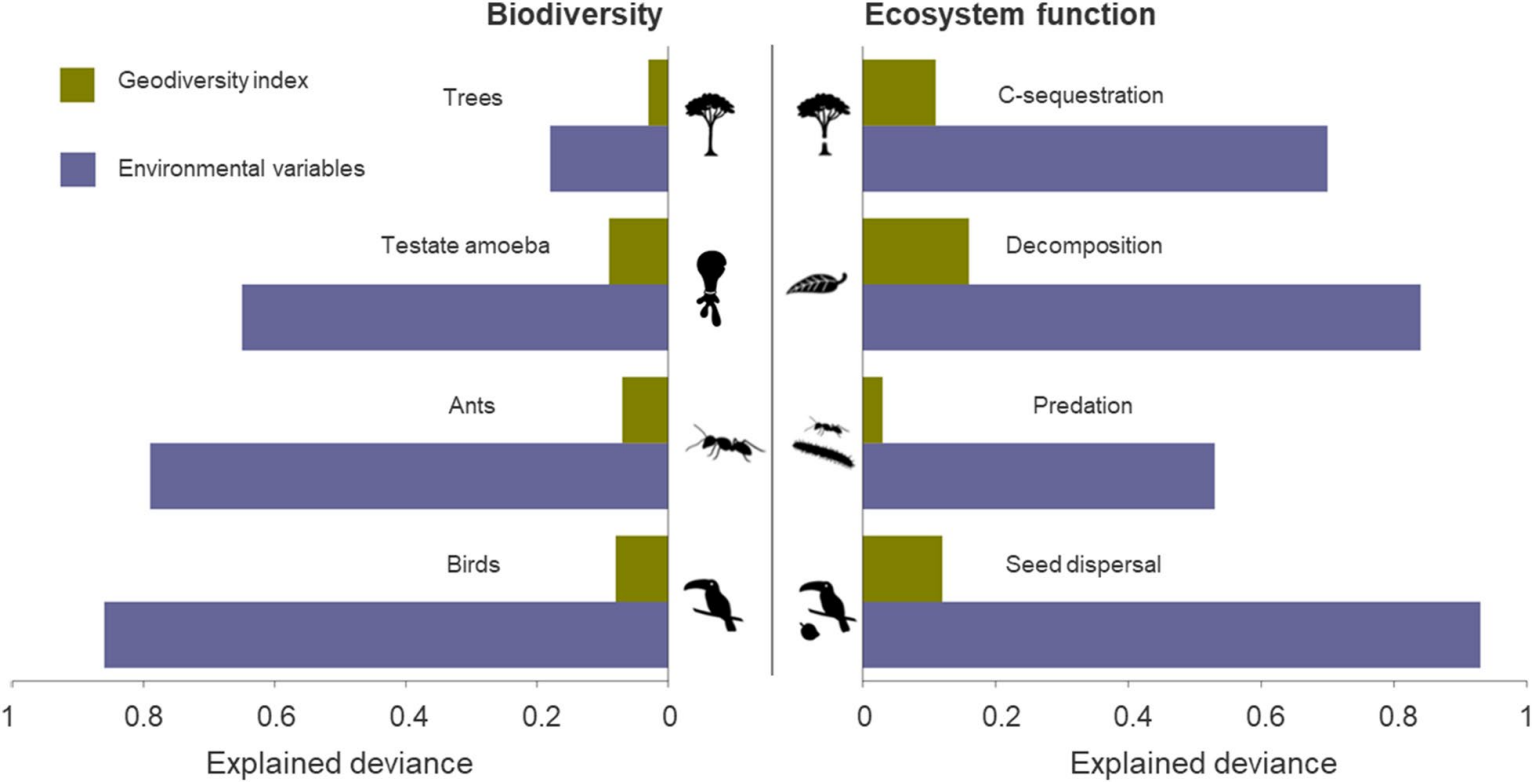
Explained deviance of species diversity (left) and ecosystem functions (right) by a geodiversity index as well as environmental variables. Models using environmental variables are based on three predictors accounting for conditions and resources of the three groups climate, habitat, and soil, while models using the geodiversity index consider the summed spatial diversities of the same three predictors. The predictor sets are selected individually for each response (depicted in Fig. 2) and are hold equal for both models based on the geodiversity index as well as environmental variables (see also Extended Data Figs. E1 and E2).

**Extended Data Figure E1:**
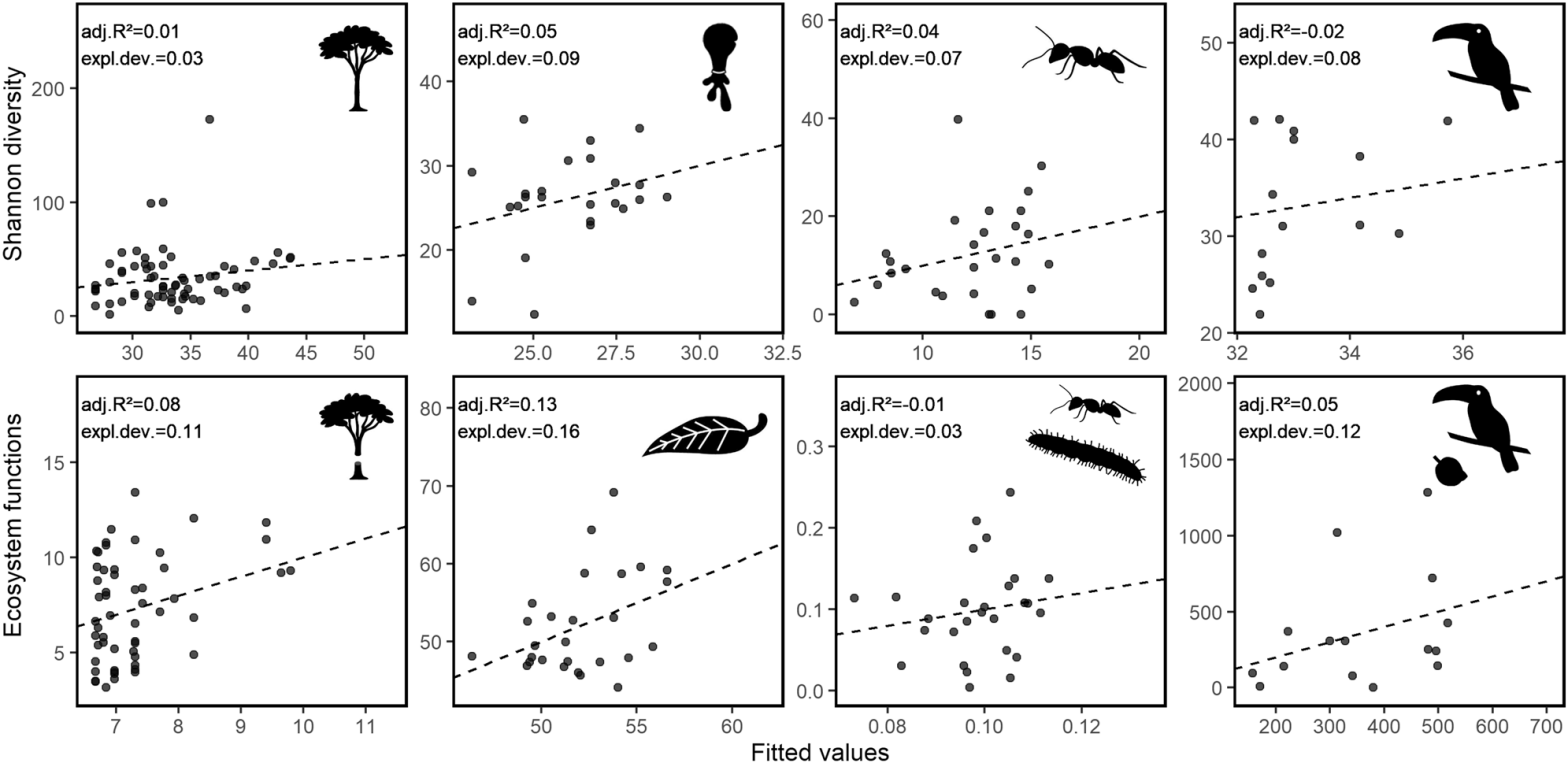
Effects of the geodiversity index on species diversity and ecosystem functions. The index of geodiversity is defined as the sum of Shannon diversity of three selected environmental predictors considering climate, habitat, and soil (see Supplementary Fig. S1). For each sampling plot, fitted (x-axis) versus observed (y-axis) values of species diversity of four taxa (trees, testate amoebae, ants, and birds; top) and related ecosystems functions (C-sequestration, decomposition, predation, and seed dispersal; bottom) are represented derived from generalized additive models (GAMs). The dashed line indicates the 1:1 line. The goodness of fit is depicted by the adjusted R^2^ and the explained deviance. Legend is shown in Fig. 2. The plant and animal images used in the figure were provided by Cordula Mann and Christiane Enderle.

**Extended Data Figure E2:**
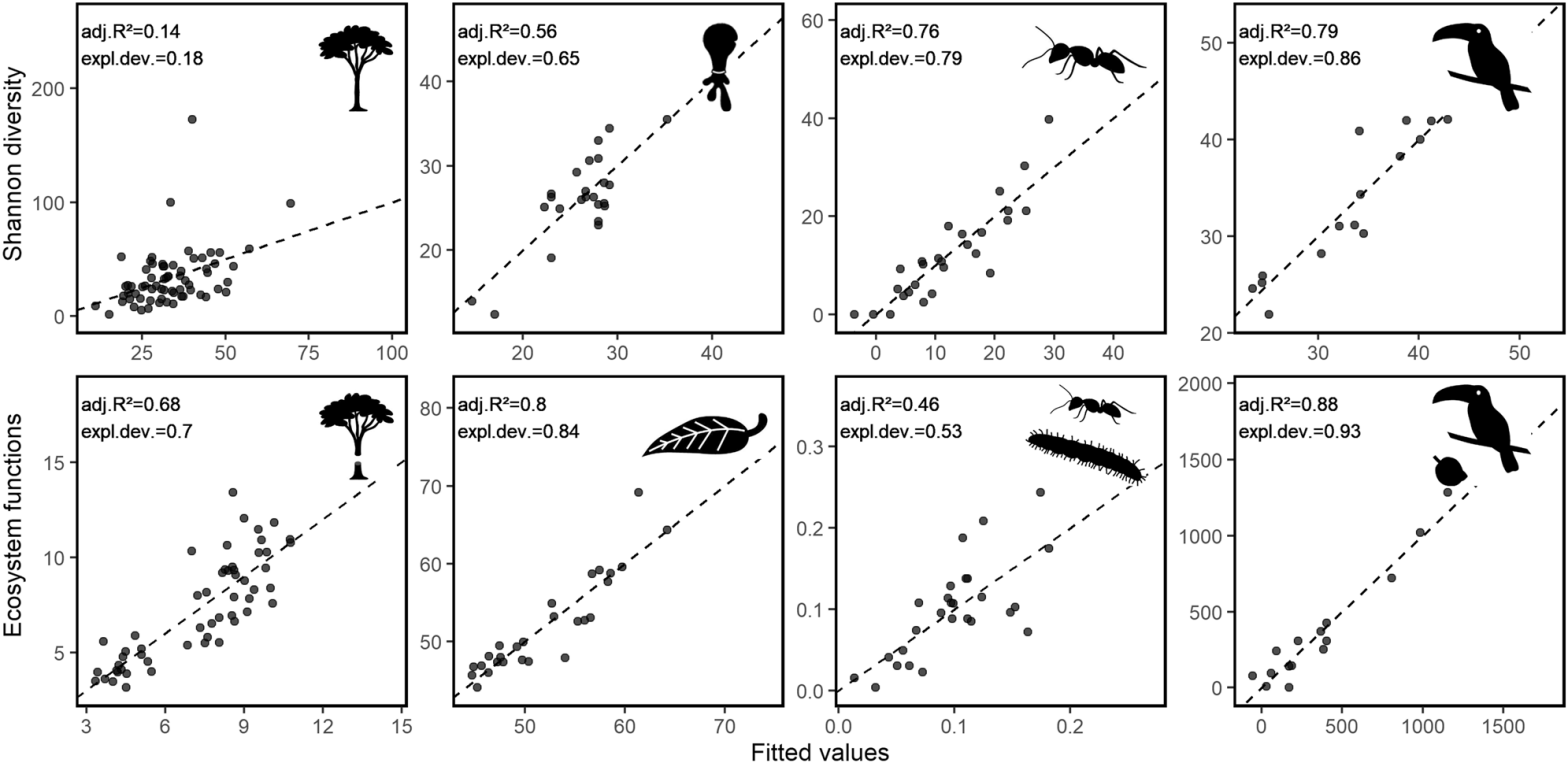
Effects of three selected environmental variables on taxon Shannon diversity and ecosystem functions. The three predictors account for environmental conditions and resources of the three groups climate, habitat, and soil. Further explanation is given in Extended Data Fig. E1 and Fig. 2. The plant and animal images used in the figure were provided by Cordula Mann and Christiane Enderle.

First, we compared the extent to which the geodiversity index and environmental variables were associated with species diversity and ecosystem functions using GAMs (generalized additive model, Fig. 1, see also Extended Data Figs. E1, E2, Extended Data Tables E3, E4, E5). Contrary to the expectation, we found that the geodiversity index explains only a very limited amount of the variation of species diversity and ecosystem functions along the elevational gradient. In contrast, models based on selected environmental conditions and resources explain most of the variation in species diversity and associated ecosystem functions (Fig. 1).

Our findings contradict the results of previous studies which successfully linked geo- and biodiversity^35,36^. On closer inspection however, only a few of the previous studies rely on geodiversity indices only (Supplementary Table S1). Most of them used mixed sets of predictors comprising both, geodiversity features and indices, and environmental variables considering different conditions and resources (e.g., climate, hydrology, soil, habitat, topography). For instance, ref.^10^ combined geodiversity indices with levels of potential radiation (climate) and NDVI (Normalized Difference Vegetation Index; habitat) to explain biodiversity. In many studies that used such a mixed set of predictors, environmental conditions and resources had higher predictive power than geodiversity indices to explain biodiversity across taxa and scales^5,34,37^.

A reason for the relatively poor performance of geodiversity indices might be the information loss by aggregating fine-grain resource data into lower resolution compound metrics. Different predictors with reversed patterns might cancel each other out in geodiversity compound indices^1^. Secondly, scale mismatches might impact the predictive performance of geodiversity. In general, the combination of environmental variables should match the targeted measurement scales at which the diversities of taxa and ecosystem functions have been sampled. In our study, these measurement scales varied depending on the different taxonomic groups studied and the associated ecosystem functions due to differences in activity ranges among the taxa (Methods). On the other hand, the used environmental data varied in their spatial resolution, mainly due to the fact that we used mostly freely available data at a certain spatial resolution (Table 1) which is a common practice in geo-biodiversity studies^38^. While there is only a small mismatch in the case of models using environmental variables (e.g., comparing tree Shannon diversity measured within an area of 400 m^2^ with the values of environmental predictors at 30 m spatial resolution), the mismatch increases when calculating spatial diversity measures from these environmental variables. In detail, the spatial scale of the environmental data in our study is in a resolution of 6–30 m and for the calculation of geodiversity the diversity measures are calculated in a 3 pixel × 3 pixel environment (320–8100 m^2^) which differ from the measurement scales of the investigated taxa and ecosystem functions (400–10,000 m^2^). In addition, this limitation resulted in geodiversity indices that in some cases combined the spatial variation of environmental variables calculated within different areas. This is a limitation of our study and needs to be addressed in future research as it is a common practice in geodiversity studies to combine remote sensing products such as digital elevation models at only moderate spatial resolution with local field data at finer resolution^35,39^. But as stated in ref.^26^ smaller mismatches between geo- and biodiversity can be tolerated and, furthermore, the knowledge that the plots have been chosen to be representative of their surrounding environment also counteracts this limitation.

Despite these limitations and recommendations, our findings suggest that geodiversity computed as a single compound index is not able to model species diversity and ecosystem functions in the here studied highly diverse tropical mountain ecosystem. This is in line with the findings in ref.^38^, that geodiversity indices better work as a surrogate for common than rare species, which often occur in biodiversity hotspot areas. By contrast, we showed that environmental variables are fundamental to conservation planning, as they imply that the availability of specific environmental conditions and resources, rather than surrogate geodiversity indices, are required to accurately predict species diversity and ecosystem functions.

### Relationships of environmental variables with species diversity and ecosystem functions

**Figure 2:**
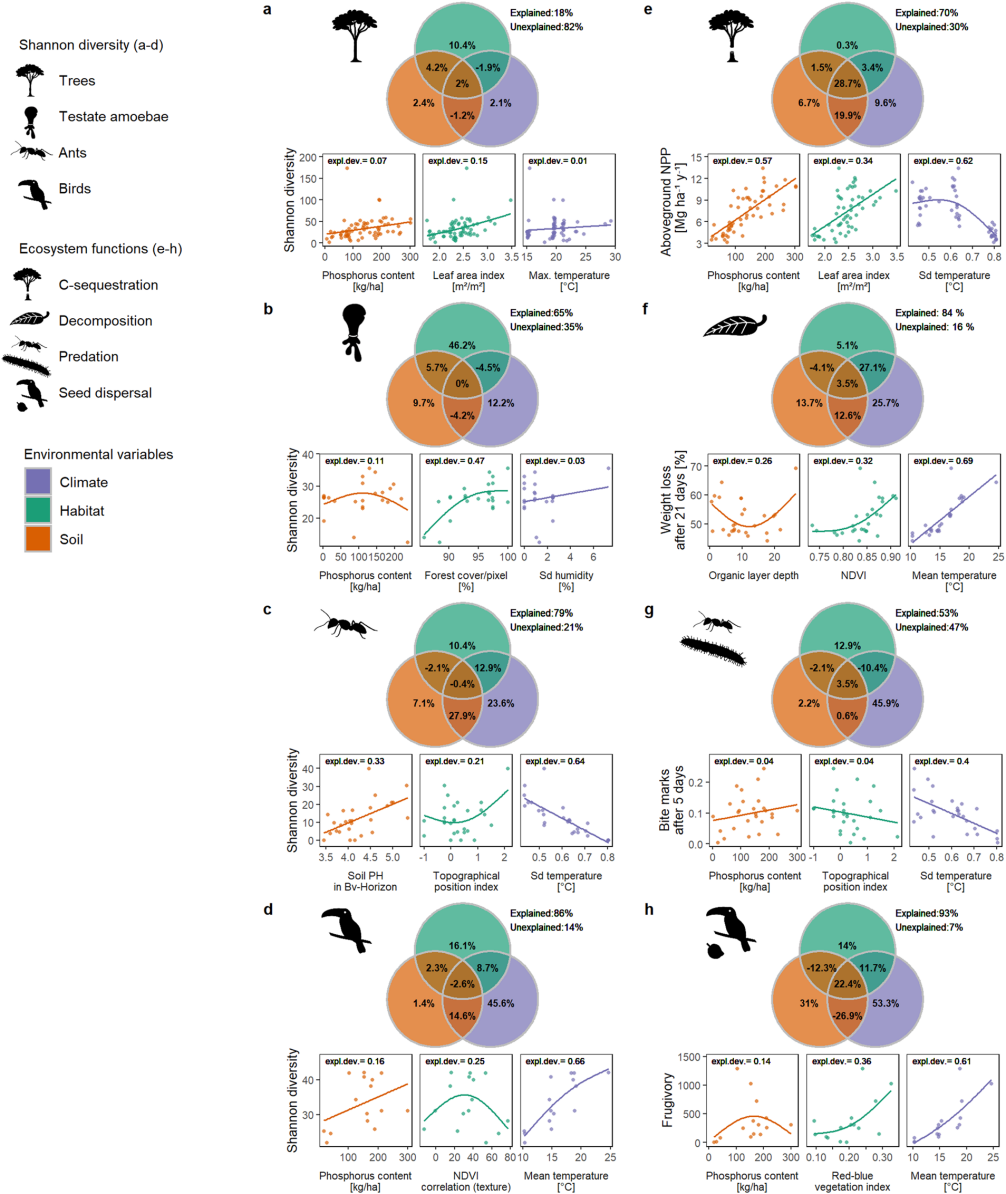
Relative importance of the three environmental variables accounting for conditions and resources from climate, habitat, and soil in explaining species diversity of four taxonomic groups (**a**–**d**) and related ecosystem functions (**e**–**h**). Upper panels: We conducted variation partitioning (VP) based on generalized additive models (GAM). Venn diagrams show the pure proportions explained exclusively by each predictor, and their share of explained deviance (expl. dev.) due to overlapping effects of pairs of predictors or all three predictors. A negative value indicates that the explained deviance by the predictor overlap is less than that explained separately by the predictors^62^. Lower panels: Dots present the response of the three selected predictors (same as in the upper panel) against Shannon taxon diversity as a measure of species diversity and the associated ecosystem functions. Lines depict the respective univariate GAM smooth functions with the corresponding explained deviance indicated. NDVI: Normalized Difference Vegetation Index, sd: standard deviation of air temperature and air humidity over time. The reader may refer to the Methods section and the Supplementary Methods for the individual selection procedure of the three predictors for each response. The plant and animal images used in the figure were provided by Cordula Mann and Christiane Enderle.

The selected environmental predictors accounting for climate, habitat, and soil variables revealed broad-scale relationships with species diversity for most taxa (Fig. 2a–d, lower panels) and ecosystem functions (Fig. 2e–h, lower panels). In the univariate models, climate conditions explained the patterns in species diversity and ecosystem functions on average better than habitat and soil predictors. In addition, variation partitioning of multivariate models showed that the most important predictors considering climate, habitat and soil differed among taxa and ecosystem functions (Fig. 2, upper panels). Conditions of air temperature were the main driver of animal diversity and ecosystem functions, whereas habitat conditions gained in relevance for plants and soil-associated taxa and functions. While tree diversity was best explained by habitat but with little explanatory support, C-sequestration was strongly related to climate, but at high shared explained variance with soil and habitat predictors. The diversity of the least mobile taxon, testate amoebae, was best explained by habitat. An increase in the diversity of testate amoebae was positively related to an increasing forest coverage which is associated with lower climatic stress^40^. Less important predictors for the diversity of testate amoebae were soil P as a key food resource^40^, and humidity^41^. Litter decomposition was best explained by climate, and in particular an increase in mean annual temperature (MAT). Litter decomposition is known to be related to the soil microbiome^42^ whose activity is positively related to MAT^43^. An increase in ant diversity and ant predation was best explained by climate, i.e., a low seasonal variability in air temperature. Ant diversity is generally positively related to enhanced temperatures^44,45^. Because ants with specifically narrow optimum temperature ranges are mostly occurring in the tropics, they particularly profit from low temperature fluctuations^46^. Bird diversity and seed dispersal by birds were positively related to MAT. This relationship corroborates findings on the global scale^47^. The importance of MAT for seed dispersal is similar to the effect on bird diversity itself, which points to more dispersal of seeds under increased temperatures. Furthermore, bird diversity showed a hump-shaped relationship to habitat, i.e., a spatial texture metric of the NDVI, confirming that species diversity is positively related to the number of niches in heterogeneous habitats^48^. The relationships between species richness and ecosystem functions with environmental conditions and resources found in this study are largely consistent with the patterns found in other tropical mountain systems^49,50^ such as Kilimanjaro^51^.

### Relevance of scale, identity, and complementarity of geodiversity for biodiversity and ecosystem functions

So far, most evidence for a general relationship between geodiversity, biodiversity and ecosystem functions is based on global analyses^2^ (but see also refs.^34,37^). Based on these studies, geodiversity has been proposed to be in principle a useful surrogate of biodiversity for conservation planning on the global scale^4,37^. Yet, our results revealed that species diversity and ecosystem functioning cannot be inferred from a geodiversity index on the regional spatial scale within a biodiversity hotspot. Instead, we found that the combination of individually selected environmental conditions and resources was much more indicative and thus, is needed to reveal associations between biodiversity and its environment on regional spatial scales. Geodiversity approaches that work well on a global scale are apparently of relatively little value for conservation management that must be implemented on the regional scale.

Our findings are analogous to those from biodiversity-ecosystem function (BEF) research where trait values are often more relevant than trait diversity to understand the role of biodiversity for ecosystem functioning at regional scales^52–54^. Similarly, a geodiversity index does not hold as an all-in-one solution to predict species diversity and ecosystem functioning. Instead, the individual environmental conditions and resources matter—and they differ between the different components of biodiversity and ecosystem functions. Both, trait identity and complementarity are needed to predict ecosystem functions from biodiversity^52–54^. This analogy suggests that research linking geo- with biodiversity and ecosystem functions might go through a similar progression of conceptual and methodological refinement as BEF research. Consequently, it must be also acknowledged, that from a conservation perspective, the here targeted species diversity is always a simplification of overall species composition. Species composition, and thus changes in species identity, are therefore often more informative and thus relevant to the conservation of biodiversity and functions^55–58^. Since the Shannon index is limited in surrogating the biotic and abiotic composition of ecosystems, we suggest that future studies should test geodiversity-biodiversity relationships focusing on the biotic and abiotic composition of environments instead of their diversity. This could be realised, i.e. using techniques of ordination to link the composition of taxa and their environment in a distance-based framework^58–61^.

## Conclusion

Our study identifies four key research gaps that future geo-biodiversity approaches should address and test with comparative studies. First, geodiversity indices seem to be better predictors of biodiversity in simple than in complex ecosystems given the selection and availability of key conditions or resources could be more important than the composition of different conditions and resources for biodiversity and functionality in these systems^34,37^. Second, the relevance of spatial scale, i.e., at larger spatial and temporal scales geodiversity indices might be more relevant than the identity of specific environmental variables given that it provides higher flexibility depending on the components of biodiversity and functions that are studied^63^. Third, the need to consider species identity in geodiversity studies, e.g., by testing the relationship between species and environmental composition instead of diversity. Fourth, the tailored selection of environmental conditions and resources is important as these affect the extent to which biodiversity and ecosystem functions can be predicted^34,37^. Our study provides new insights into how combinations of environmental variables, rather than geodiversity indices, shape species diversity and ecosystem functions at regional scales. At such spatial scales, both the selection and the combination of environmental variables can increase our mechanistic understanding and support conservation management of biodiversity and ecosystem functions.

## Methods

### Study area

**Figure 3:**
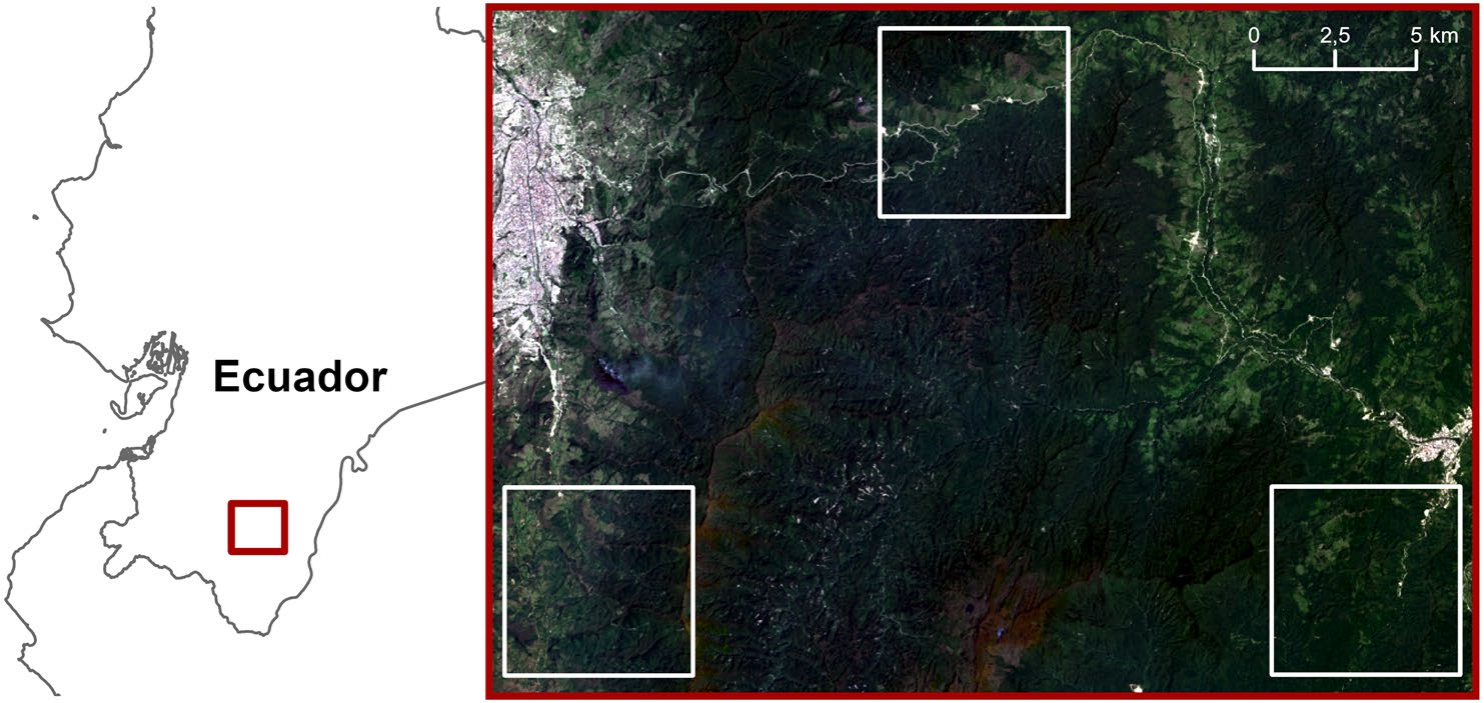
Location of the study area in the southern part of Ecuador (left) and its three main elevational levels from 3000 to 1000 m a.s.l. (white boxes from left to right). Map created in QGIS 3.14.16-Pi (https://qgis.org/). Background imagery (right): Landsat-8 true-color composite (in courtesy of USGS, https://earthexplorer.usgs.gov/).

We exemplify our analysis on the mountain rainforest of south-eastern Ecuador that belongs to the biodiversity hotspot of the tropical Andes which is globally most important for conservation support^64^. We use data of a unique ecosystem, the mountain rainforest of the eastern Andean slopes^65^. This is essential to avoid confounding effects from multiple biogeographic units, as it is the case in most other studies on the relation of geo- and biodiversity. The field data were collected between 2007 and 2017 along an elevational gradient between 1000 and 3000 m a.s.l. (Fig. 3) with study sites equally distributed along the gradient^66–68^.

### Species diversity and ecosystem functions

We assessed the species diversity of four taxa and four associated ecosystem functions using standardized methods. The details of the sampling procedure of each taxon and function have mostly been described in their original publications. We sampled species diversity data of trees^40,69^, testate amoebae^70,71^, ants^72^ and birds^73^. For ants, species abundance data from two field campaigns were pooled and for birds, nine point count records per plot were pooled to obtain the bird species abundance of all species and individuals per plot. Species abundance for trees and testate amoebae was measured based on distinct samples. To compare the Shannon diversity of the four taxa in a standardized way, we used a rarefaction and extrapolation framework based on sample coverage, implemented in the R package iNEXT^74,75^. In this framework, it is statistically valid to extrapolate the observed Shannon diversity values to the maximal sample coverage (the asymptote of the rarefaction and extrapolation curve). We therefore set the extrapolation ratio for the calculation of the standardized species diversity values to one, corresponding to a complete sample coverage for all taxa^76^.

Four important ecosystem functions that are associated with the four taxa were measured in the study area. C-sequestration was observed as the aboveground net primary production^77^, deduced from annual wood production and annual fine litter production (tree diversity/C-sequestration, partial overlap of plots, Pearson r = 0.35, p < 0.01, n = 52). The decomposition of soil organic matter was measured using a Tea Bag experiment^78^ reflecting the weight loss of tea bags after 21 days (testate amoebae diversity/decomposition, distinct plots). Predation was assessed using the number of bite marks of ants on artificial caterpillars^72^ (ant diversity/predation, Pearson r = 0.40 p < 0.05, n = 27). Seed dispersal was measured as the number of observed seed removal events of frugivorous birds on fleshy fruited plants^79^ (bird diversity/seed dispersal, Pearson r = 0.62, p < 0.01, n = 15).

### Environmental variables

In this study we investigated a set of 13 environmental variables based on conditions and resources within the three groups climate, habitat and soil (Table 1). We did not include geology which has often been used in other geodiversity research studies due to the geological homogeneity of our study area^66^. The predictors of the three groups were available as spatial datasets or were spatially predicted with varying spatial resolution (see Table 1). The following predictors were considered for the three groups:

Climate: We used climate stations in combination with a Landsat-8 image classification (recorded on 20/11/2016, see also ref.^77^) to derive gridded forest temperature and humidity data at a spatial resolution of 30 m following previous approaches^80,81^. We calculated four climatic predictors: the annual mean, maximum and standard deviation of mean monthly air temperatures over the year as well as the mean annual relative air humidity.

Habitat: Based on the pre-processed Landsat-8 scene, we calculated the Normalized Difference Vegetation Index (NDVI) and its textural metric ‘correlation’ using all directions in the ‘glcm’ package^82^ in R as well as the Red-Blue Difference Vegetation index (RBVI, see refs.^77,83^) and the forest cover per pixel in percentage. These habitat variables are known to be related to habitat structure and productivity in the study area^77,83^. In addition, we used the Sentinel-2 product Leaf Area Index (LAI) available at 10 m spatial resolution. To characterize topography, we calculated the Topographical Position Index (TPI) based on an airborne high-spatial-resolution digital elevation model from the SIGTIERRAS campaign^83^.

Soil: Due to the lack of spatial soil data products at a moderate spatial resolution, we modeled and spatially predicted a suite of soil variables sampled in the study area^84^. These soil variables were related to phosphorus and nitrogen availability and pH (in H_2_O) in the mineral soil horizon. We used multi-linear models with a stepwise predictor selection (forward and backward) using spatial predictors (Extended Data Table E1). Only three models explained more than 45% in organic layer depth, Phosphorus content in Ah-horizon and pH in Bv-horizon. The explained variance of their models ranged between 45 and 69% (Extended Data Table E2). Their spatial predictions were subsequently used as soil predictors.

### Environmental variables and geodiversity indices

Our study targets to compare models of species diversity and ecosystem functions using combinations of environmental predictors grouped within climate, habitat, and soil variables with models using a single geodiversity compound index which is defined as the summed spatial diversity of the same selected environmental variables. For this purpose, from each group (climate, habitat, and soil) one condition or resource has been selected as predictor. The choice of a predictor within each group (e.g., temperature, rainfall etc. out of climate) was guided by the findings of prior studies (Extended Data Table E3, Supplementary Methods) and the resulting combination of environmental variables, thus, comprises the requirements that the corresponding organisms need for their establishment and survival. A second criterion was the collinearity present in the predictor space. We changed the predictor combination if the correlation between two or more predictors were greater than r = 0.6. Whenever present, different statistics such as mean, maximum, and standard deviation of the climate group have been compared and the environmental variables with the best performance concerning the Akaike information criterion of the corresponding model fit was chosen. For all response variables, the pixel values of the selected environmental variables were used that overlapped with either the point or shape geometries of the corresponding plots. The resulting predictor set, thus, assess the central tendency of three environmental factors at each plot.

The geodiversity index, in contrast, is computed as the summed spatial diversity of the same three environmental factors measured within each plot and its surrounding and follows the recent plea to consistently use diversity indices commonly applied in biodiversity studies^7^. For this purpose, we classified the spatial grids of the three selected predictors into five classes using intervals following the Fisher style^85^ in the ‘classInt’ R package^86^. After the classification, the central pixel of each plot and in addition all adjacent pixels were extracted. This resulted in nine pixels per plot. For these pixels, the frequency of all present classes was calculated. From an ecological perspective each class was used analogous to a present species at the corresponding plot and their frequency was used as their abundance. For the resulting abundance matrix of the three predictors, Shannon diversity was calculated using the diversity function of the ‘vegan’ package^87,88^. This results in three predictors addressing the spatial variation of the chosen environmental variables. As in some ecological studies where the diversity of multiple taxa has been summed or averaged to study multi-taxa diversity^51^, we here treated each of the three predictors like a single taxon. Thus, analogously to multi-taxa approaches, we calculated the geodiversity index by summing up the spatial diversity of the three environmental variables (for further explanation see also Supplementary Fig. S1).

### Statistical analyses

We used generalized additive regression models (GAMs) to model species diversity, and ecosystem functions. To address the (non-)linear relationships between the index of geodiversity, species diversity and ecosystem functions, univariate GAMs were used. Multivariate GAMs were used to address the relationships to the three environmental variables considering conditions and resources of the three groups climate, habitat, and soil. For all models, GAMs were calibrated using a Gaussian error distribution in the model fitting based on the exploration of residuals and the generalized cross-validation score of the models (GCVS). Non-linear relationships were considered using cubic regression splines with a degree of freedom permitted to vary between one and three. GAMs however maintained the flexibility to model linear relationships wherever present. We performed all GAM analyses using the ‘mgcv’ package in R^89^. The model performance of combined environmental variables and the geodiversity index among all models was compared (Fig. 1).

For the combination of environmental variables, we further explored the driving conditions and resources within climate, habitat and soil variables considering their pure and shared proportions in explaining species diversity and ecosystem functions. For that purpose, we used Variance Partitioning (VP) implemented in the ‘modEvA’ R package^90^ in combination with GAMs. All environmental variable combinations from single to three predictors were considered, resulting in 7 different GAMs (3 models with single predictors, three models with two predictors and one model with three predictors). Model parameters have been fixed as in the model using all three climate, habitat, and soil predictors to enhance model comparability.

## Extended data

See Extended Data Figs. E1, E2 and Tables E1, E2, E3, E4 and E5.

**Extended Data Table E1:** Spatial predictors to derive the three soil variables (shown in Table 1).

Predictor of soil variables	Source	Calculation/references
Digital elevation model (DEM)	Aster image	USGS
Slope	Aster image	Based on DEM, in degree
Topographical position index (TPI)	Aster image	After ref.^93^, with a surrounding of 11 pixels
Mean temperature	Weather stations, Landsat classification	After ref.^44^
Canopy water content (Canopy water)	Sentinel 2	S2 SNAP (Sentinel Application Platform) Toolbox Biophysical Processor^54^
Foliar N content (Foliar N)	Landsat-8	From ref.^43^

The spatial predictors were used to model the soil variables and derive prediction maps. Equations shown in Extended Data Table E2.

**Extended Data Table E2:** Multi-linear model equations with stepwise predictor selection and model fit to derive the three soil variables of Table 1.

Predicted soil variables	Model equation	Adjusted R^2^
Organic layer depth in cm (organic layer depth, n = 53)	OLD ~ FoliarN + Canopy water + Slope + TPI	0.45
Phosphorus content in Ah-Horizon in kg/ha (Phosphorus; n = 43)	Phosphorus ~ FoliarN + Canopy water + DEM	0.69
pH in Bv-Horizon (pH; n = 50)	pH ~ Mean temperature + TPI	0.67

All initial predictors are shown in Extended Data Table E1. The number of soil samples is given by n in brackets. All predictors were resampled to 30 m spatial resolution.

**Extended Data Table E3:** Selected environmental variables used as predictors in generalized additive models for modeling species diversity for four taxa and associated ecosystem functions.

	Climate			Habitat			Soil			References
	Predictor	Mean	Sd	Predictor	Mean	Sd	Predictor	Mean	Sd	
**Shannon diversity**										
Trees (n = 67)	Max temperature	19.30	3.07	LAI	2.38	0.34	Phosphorus	137.98	72.59	^95^
Testate amoebae (n = 24)	Sd humidity	1.43	1.59	Forest cover/pixel	0.94	0.04	Phosphorus	106.47	74.9	^96^
Ants (n = 27)	Sd temperature	0.60	0.11	TPI	0.31	0.71	pH	4.24	0.52	^45,83^
Birds (n = 15)	Mean temperature	15.89	3.94	NDVI correlation	34.17	23.91	Phosphorus	147.92	75.58	^73,83^
**Ecosystem functions**										
C-sequestration (n = 54)	Max temperature	0.63	0.14	LAI	2.43	0.36	Phosphorus	135.45	71.86	^40^
Decomposition (n = 27)	Mean temperature	15.48	3.19	NDVI	0.84	0.04	Organic layer depth	10.55	6.82	^43^
Predation (n = 27)	Sd temperature	0.60	0.11	TPI	0.31	0.71	Phosphorus	132.94	70.05	^72^
Seed dispersal (n = 15)	Mean temperature	15.89	3.94	RBVI	0.20	0.07	Phosphorus	147.92	75.58	^79^

Predictors were grouped within climate, habitat, and soil conditions and resources, respectively. References highlighting their use as predictors of species diversity are indicated (see also Supplementary Methods). The number of samples of each response is given by n in brackets.

**Extended Data Table E4:** Generalized additive model results using a geodiversity index as a single predictor.

Response	Parametric coefficients (Intercept)				Approximate significance of smooth terms					Model fit	
	Estimate	Std. Error	t value	Pr( >|t|)	edf	Ref.df	F	p-value	EDF total	adjR^2^	Exp. Dev
**Shannon diversity**											
Trees	33.42	3.1	10.77	0	1	1	1.88	0.18	2	0.01	0.03
Testate amoebae	26.07	1.07	24.33	0	1	1	2.27	0.15	2	0.05	0.09
Ants	12.23	1.81	6.74	0	1	1	1.95	0.17	2	0.04	0.07
Birds	33.17	1.88	17.65	0	1.4	1.64	0.34	0.72	2.4	-0.02	0.08
**Ecosystem functions**											
C-sequestration	7.29	0.35	20.52	0	1.85	1.98	2.75	0.07	2.85	0.08	0.11
Decomposition	52.07	1.13	46.11	0	1	1	4.91	0.04	2	0.13	0.16
Predation	0.10	0.01	8.64	0	1	1	0.67	0.42	2	-0.01	0.03
Seed dispersal	359.40	93.99	3.82	0	1	1	1.75	0.21	2	0.05	0.12

A Gaussian family was used for all models. Exp. Dev. = explained deviance.

**Extended Data Table E5:** Generalized additive model results using selected environmental variables as predictors.

Response	Parametric coefficients (intercept)				Approximate significance of smooth terms (predictors)							Model fit	
	Est	Std. error	t value	Pr ( >|t|)	Climate	P-value climate	Habitat	P-value habitat	Soil	P-value soil	EDF total	adjR^2^	Exp. Dev
**Shannon diversity**													
Trees	33.42	2.90	11.52	0	Max temperature	0.20	LAI	0.02	Phosphorus	0.20	4.27	0.14	0.18
Testate amoebae	26.07	0.73	35.69	0	Sd humidity	0.03	Forest cover/pixel	0.00	Phosphorus	0.16	5.78	0.56	0.65
Ants	12.23	0.91	13.46	0	Sd temperature	0.00	TPI	0.02	pH	0.01	4.86	0.76	0.79
Birds	33.17	0.85	39.19	0	Mean temperature	0.00	NDVI correlation	0.03	Phosphorus	0.44	5.59	0.79	0.86
**Ecosystem functions**													
C-sequestration	7.29	0.21	34.74	0	Max temperature	0.00	LAI	0.53	Phosphorus	0.00	4.67	0.68	0.70
Decomposition	52.07	0.54	96.12	0	Mean temperature	0.00	NDVI	0.08	Organic layer depth	0.00	5.74	0.80	0.84
Predation	0.10	0.01	11.86	0	Sd temperature	0.00	TPI	0.02	Phosphorus	0.32	4.02	0.46	0.53
Seed dispersal	359.40	33.14	10.84	0	Mean temperature	0.00	RBVI	0.01	Phosphorus	0.00	6.94	0.88	0.93

A Gaussian family was used for all models. Est. = Estimate, Exp. Dev. = explained deviance.

## Supplementary Information

Supplementary Information.

## Data Availability

Data on species abundance of taxonomic groups and ecosystem functions will be made available in the data warehouse^91^ under www.TropicalMountainForest.org.

## Code availability

The R code and the datasets generated and analysed within the current study are available from the corresponding authors on reasonable request.
