# Supplementary material for: Biodiversity and ecosystem functions depend on environmental conditions and resources rather than the geodiversity of a tropical biodiversity hotspot
Source: Sci Rep. 2021 Dec 31;11:24530. doi: 10.1038/s41598-021-03488-1 (PMC8720099; doi:10.1038/s41598-021-03488-1)
Supplement: Supplementary file 1 — Supplementary Information. [file 41598_2021_3488_MOESM1_ESM.docx]

**Supplementary information:**

**Biodiversity and ecosystem functions depend on environmental conditions and resources rather than the geodiversity of a tropical biodiversity hotspot**

**Christine I.B. Wallis*, Yvonne C. Tiede, Erwin Beck, Katrin Böhning-Gaese, Roland Brandl, David A. Donoso, Carlos I. Espinosa, Andreas Fries, Jürgen Homeier, Diego Inclan, Christoph Leuschner, Mark Maraun, Katrin Mikolajewski, Eike Lena Neuschulz, Stefan Scheu, Matthias Schleuning, Juan P. Suárez, Boris A. Tinoco, Nina Farwig, Jörg Bendix**

**DOI: 10.1038/s41598-021-03488-1**

***Corresponding author:**

Christine I. B. Wallis

Département de biologie

Université de Sherbrooke

Sherbrooke, Québec J1K 2R1, Canada

christine.wallis@USherbrooke.ca


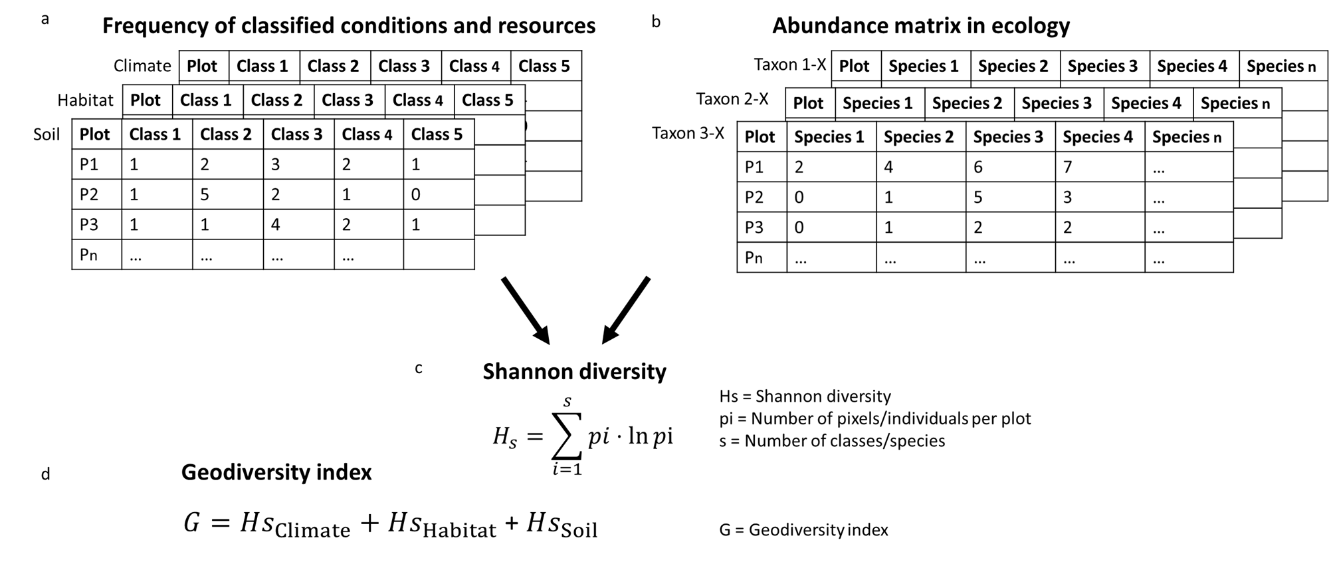


**Supplementary Figure S1** **| Derivation of a geodiversity index analogous to taxonomic diversity.** a. Abundance matrices of classified environmental conditions and resources from the three groups climate, habitat, and soil. For each response one predictor was chosen from each group (see Extended Data Table E3 and Supplementary Methods). For all selected environmental conditions/resources a class size of five was chosen. The number of individuals per plot equals always nine due to the extraction of nine pixels per plot. b. Abundance matrices of commonly used species diversity approaches. c. The Shannon-Wiener diversity index equation was used for both taxonomic species abundance and class abundance within the selected climatic, habitat, and soil conditions or resources. The latter addresses the diversity of environmental variables in each group. d. For the geodiversity index, the Shannon diversity of the three selected environmental variables was summed.

**Supplementary Table S1 | Geodiversity predictors and biodiversity responses and indices used in different geodiversity studies.**

|  | Aim | Geodiversity predictor | Geodiversity index | Biodiversity measure | Reference |
| --- | --- | --- | --- | --- | --- |
| Geodiversity only | Geo-conservation | Geological (rocks, minerals, fossils), geomorphological (land form, physical processes) and soil features | Assemblages, relationships, properties | - | ^1^ |
|  | Geo-conservation | i) Stratigraphical, palaeontological, sedimentary, igneous, metamorphic, mineralogical, economical, geochemical, seismical, structural, palaeogeographical, cosmogenic, geothermal, geocryological, geomorphological, hydrological and hydrogeological, engineering, radiogeological, neotectonical, pedological (soil), and geohistorical features | Sum of feature numbers, total quantity complex geosites / total geosites | - | ^2^ |
|  | Geodiversity resource management and exploitation | Geological, geomorphological, paleontological, pedological, mineral occurrence features | Sum of feature numbers | - | ^3^ |
|  | Geodiversity for natural heritage planning | Soil, geology, hydrography and relief features | PCA on feature numbers | - | ^4^ |
|  | Geodiversity assessment | Morphometric, morphoclimatic, geological classes | Patch Richness Density, Shannon’s Diversity Index, Shannon’s Evenness Index, Simpson’s, Diversity Index and Simpson’s Evenness Index | - | ^5^ |
|  | Geodiversity assessment with remote sensing | Geological, geomorphological and hydrological features | Sum of feature number | - | ^6^ |
| Geodiversity & biodiversity | Explaining biodiversity by geodiversity | (i) No of soil, bedrock and hydrological features, (ii) topographic variables, (iii) landscape variables (habitat type, land cover, spectral heterogeneity) | (i) Sum of feature numbers (ii) mean, sd (iii) No of patches | vascular plant species richness | ^7^ |
|  |  | (i) Three geodiversity (geological, geomorphological and hydrological units) variables, (ii) six climate and topography features | (i) Sum of feature numbers (ii) mean, sd, range | vascular plant species richness | ^8^ |
|  |  | Components (climate, topography, geology and hydrology) and resources (Water, energy, space, nutrients) of geodiversity | Geodiversity compound index (resource availability, temporal and spatial variation) | Diversity of mammals, birds, amphibians | ^9^ |
|  |  | Geomorphologic features | Descriptive | Diversity of plants and animals | ^10^ |
|  |  | (i) Geodiversity: landforms, soils, hydrological and geological features (ii) climate, commonly used topographic metrics, land-cover variety and human population | (i) Number of features (ii) Mean values, number of land cover types | Species richness of terrestrial native and alien vascular plants | ^11^ |
|  |  | (i) Geodiversity: Geological, soil, hydrological and topographical features, (ii) GTOPO30, NPP MODIS, climate | (i) Combined geodiversity (GD) index value 1-20, (ii) levels | Mountain vascular plant species diversity | ^12^ |
|  |  | (i) Geodiversity (geological, geomorphological and hydrological richness), (ii) climate, (iii) spatial variables | (i) Number of features, (ii) median, (iii) principal coordinates of neighbor matrices (PCNMs) | Vascular plant species richness | ^13^ |
|  |  | (i) Geodiversity (topography, soil, geology), (ii) climate | (i) Shannon entropy also spatial (ii) means | Bird and tree diversity | ^14^ |
|  |  | Topographic geodiversity | Elevation values (sd in 5-100 km grids) | Tree biodiversity | ^15^ |
|  |  | Geodiversity (degree of stoniness, soil features) | Levels | Biocrust's composition | ^16^ |

**Supplementary Methods**

**Choice of environmental variables as predictors for models of taxon diversity and ecosystem functions.** Elevation has been widely applied as a major predictor of species diversity, surrogating the elevational changes of various conditions and resources, as i.e., a decrease with rising temperature^17^ and biotic changes such as a compositional change of plant species^18^. The diversity of many taxonomic groups is known to decrease with increasing elevation (i.e. for ants^19^ or microarthropods^20,21^). However, this relationship is affected by the scale of the study and is not valid for all taxa^3^. This has been also shown for certain taxa in the studied region^22–24^. To better understand regional changes along elevational gradient we accomplished a set of 13 spatial predictors that capture not only the elevational gradient but also consider the environmental conditions and resources within the ecosystem which might have an important effect on the distribution of species diversity of multiple taxonomic groups and their corresponding functions.

We categorized these predictors into the three groups climate, habitat, and soil. Because of the different resource requirements of the taxonomic groups studied here, we chose the environmental condition or resource within each group individually for each taxon diversity and ecosystem function. We chose three predictors for each model, with one predictor from each of the three groups. To reduce multi-collinearity between the three predictors, we removed predictor pairs with correlations higher a Pearson’s-r=0.6. The selection of predictors was guided by previous research, particularly in the same study region. In cases where no adequate reference was found, we chose the predictor which increased the explained variance the most.

We used the mean temperature of monthly averages as a climate predictor for bird diversity, seed dispersal and decomposition. Bird diversity showed a strong increase with decreasing elevation and temperature as well as its associated ecosystem function seed dispersal in prior studies within the Ecuadorian mountain forest^22,25,26^, a pattern found also on the global scale^27^. For decomposition we hypothesize a strong effect of species turnover which changes with temperature variations along the elevational gradient in the study area^22^. For ant diversity, predation, and aboveground NPP we chose the standard deviation of temperature which refers to climatic stability. The response of ectotherm species to temperature is well-documented^28^ and from prior studies in the study area, a decrease of ant species diversity with increasing elevation and thus decreasing temperature was known^22,29^. At the same time, ref.^30^, supported the climatic stability hypothesis for ant diversity indicating that a more stable climate favours higher ant diversity. Especially ectotherms, such as ants, can profit from low temperature fluctuations due to their narrow optimum temperature ranges which can be even more pronounced in the tropics, where seasonal temperature variation is generally low^31,32^. For testate amoebae diversity, we also considered climatic stability, but chose the standard deviation of relative air humidity due to the well-known relationship of testate amoebae diversity and moisture^33^. For tree species we used the maximum monthly temperature as a climate resource although prior studies show no significant relationship between tree diversity and elevation^22,24^ (but see also ^34^). Studies at other locations find that tree species diversity also increase with rainfall of the warmest month^35^.

From the habitat group, we applied various predictors. For tree diversity and aboveground NPP, for example, we chose the leaf area index (LAI) which is known to show a positive relation to diversity in prior studies^36,37^. For testate amoebae, we used the forest coverage due to its positive effect on its diversity which can be explained by its relation to low UV radiation^33^. In case of ant diversity and predation, the topographical position index was used. It addresses the different slope positions in a landscape which have been proven as an important driver of ant diversity^38^. For decomposition, bird diversity and seed dispersal, spectral indices derived from airborne remote sensing data have been used as well. The use of the Normalized Difference Vegetation Index (NDVI) is supported by various studies that underline its positive effect on litter decomposition^39^. For bird diversity, we chose the texture metric correlation based on the NDVI, which is a surrogate for habitat structure. Texture metrics have been presented and proven as suitable habitat structural predictors in prior studies^40,41^. The relationship between habitat structure and bird diversity is known for the investigated research area^40^ and in general^27^, highlighting that species diversity is positively related to an increasing number of niches in heterogeneous habitats.

For soil variables we most often used phosphorus content. Soil nutrients in general, and particularly phosphorus content have strong effects on the productivity of the forest^42^ which in turn might influence the presence of species and subsequently also functions. For decomposition, however, we used the organic layer instead according to its strong association to the decomposition rate^43^. For ant diversity, we used soil pH since Staab et al.^44^ showed that the ant community is not related to soil nutrients but strongly affected by soil pH with less diverse communities on acidic soil.

**Supplementary references**

1. Gray, M. Geodiversity and Geoconservation: What, Why, and How? *George Wright Forum* **22**, 4–12 (2005).

2. Ruban, D. A. Quantification of geodiversity and its loss. *Proc. Geol. Assoc.* **121**, 326–333 (2010).

3. Pereira, D. I., Pereira, P., Brilha, J. & Santos, L. Geodiversity Assessment of Paraná State (Brazil): An Innovative Approach. *Environ. Manage.* **52**, 541–552 (2013).

4. dos Santos, F. M., de La Corte Bacci, D., Saad, A. R. & da Silva Ferreira, A. T. Geodiversity index weighted by multivariate statistical analysis. *Appl. Geomat.* **12**, 361–370 (2020).

5. Benito-Calvo, A., Pérez-González, A., Magri, O. & Meza, P. Assessing regional geodiversity: the Iberian Peninsula. *Earth Surf. Process. Landf.* **34**, 1433–1445 (2009).

6. Hjort, J. & Luoto, M. Can geodiversity be predicted from space? *Geomorphology* **153–154**, 74–80 (2012).

7. Räsänen, A. *et al.* The role of landscape, topography, and geodiversity in explaining vascular plant species richness in a fragmented landscape. *Boreal Environ. Res.* **21**, 53–70 (2016).

8. Hjort, J., Heikkinen, R. K. & Luoto, M. Inclusion of explicit measures of geodiversity improve biodiversity models in a boreal landscape. *Biodivers. Conserv.* **21**, 3487–3506 (2012).

9. Parks, K. E. & Mulligan, M. On the relationship between a resource based measure of geodiversity and broad scale biodiversity patterns. *Biodivers. Conserv.* **19**, 2751–2766 (2010).

10. Bétard, F. Patch-Scale Relationships Between Geodiversity and Biodiversity in Hard Rock Quarries: Case Study from a Disused Quartzite Quarry in NW France. *Geoheritage* **5**, 59–71 (2013).

11. Bailey, J. J., Boyd, D. S., Hjort, J., Lavers, C. P. & Field, R. Modelling native and alien vascular plant species richness: At which scales is geodiversity most relevant?: BAILEY et al. *Glob. Ecol. Biogeogr.* **26**, 763–776 (2017).

12. Muellner-Riehl, A. N. *et al.* Origins of global mountain plant biodiversity: Testing the ‘mountain‐geobiodiversity hypothesis’. *J. Biogeogr.* **46**, (2019).

13. Tukiainen, H. *et al.* Spatial relationship between biodiversity and geodiversity across a gradient of land-use intensity in high-latitude landscapes. *Landsc. Ecol.* **32**, 1049–1063 (2017).

14. Read, Q. D. *et al.* Beyond counts and averages: Relating geodiversity to dimensions of biodiversity. *Glob. Ecol. Biogeogr.* **29**, 696–710 (2020).

15. Zarnetske, P. L. *et al.* Towards connecting biodiversity and geodiversity across scales with satellite remote sensing. *Glob. Ecol. Biogeogr.* **28**, 548–556 (2019).

16. Zaady, E., Stavi, I. & Yizhaq, H. Hillslope geodiversity effects on properties and composition of biological soil crusts in drylands. *Eur. J. Soil Sci.* ejss.13097 (2021) doi:10.1111/ejss.13097.

17. Angilletta Jr., M. J. *Thermal Adaptation*. (Oxford University Press, 2009). doi:10.1093/acprof:oso/9780198570875.001.1.

18. Malizia, A. *et al.* Elevation and latitude drives structure and tree species composition in Andean forests: Results from a large-scale plot network. *PLOS ONE* **15**, e0231553 (2020).

19. Bishop, T. R., Robertson, M. P., Rensburg, B. J. V. & Parr, C. L. Coping with the cold: minimum temperatures and thermal tolerances dominate the ecology of mountain ants. *Ecol. Entomol.* **42**, 105–114 (2017).

20. Illig, J., Norton, R. A., Scheu, S. & Maraun, M. Density and community structure of soil- and bark-dwelling microarthropods along an altitudinal gradient in a tropical montane rainforest. *Exp. Appl. Acarol.* **52**, 49–62 (2010).

21. Maraun, M., Fronczek, S., Marian, F., Sandmann, D. & Scheu, S. More sex at higher altitudes: Changes in the frequency of parthenogenesis in oribatid mites in tropical montane rain forests. *Pedobiologia* **56**, 185–190 (2013).

22. Wallis, C. I. B. *et al.* Remote sensing improves prediction of tropical montane species diversity but performance differs among taxa. *Ecol. Indic.* **83**, 538–549 (2017).

23. Fiedler, K., Brehm, G., Hilt, N., Süßenbach, D. & Häuser, C. L. Fauna: Composition and Function. in *Gradients in a Tropical Mountain Ecosystem of Ecuador* (eds. Beck, E., Bendix, J., Kottke, I., Makeschin, F. & Mosandl, R.) 167–179 (Springer, 2008). doi:10.1007/978-3-540-73526-7_17.

24. Tiede, Y. *et al.* Phylogenetic niche conservatism does not explain elevational patterns of species richness, phylodiversity and family age of tree assemblages in Andean rainforest. *Erdkunde* **70**, 83–106 (2016).

25. Santillán, V. *et al.* Spatio-temporal variation in bird assemblages is associated with fluctuations in temperature and precipitation along a tropical elevational gradient. *PLOS ONE* **13**, e0196179 (2018).

26. Quitián, M. *et al.* Elevation-dependent effects of forest fragmentation on plant-bird interaction networks in the tropical Andes. *Ecography* **41**, 1497–1506 (2018).

27. Tews, J. *et al.* Animal species diversity driven by habitat heterogeneity/diversity: the importance of keystone structures: Animal species diversity driven by habitat heterogeneity. *J. Biogeogr.* **31**, 79–92 (2004).

28. McCain, C. M. Global analysis of bird elevational diversity. *Glob. Ecol. Biogeogr.* **18**, 346–360 (2009).

29. Tiede, Y. *et al.* Ants as indicators of environmental change and ecosystem processes. *Ecol. Indic.* **83**, 527–537 (2017).

30. Nowrouzi, S. *et al.* Ant Diversity and Distribution along Elevation Gradients in the Australian Wet Tropics: The Importance of Seasonal Moisture Stability. *PloS One* **11**, e0153420 (2016).

31. Paaijmans, K. P. *et al.* Temperature variation makes ectotherms more sensitive to climate change. *Glob. Change Biol.* **19**, 2373–2380 (2013).

32. Sunday, J. M., Bates, A. E. & Dulvy, N. K. Thermal tolerance and the global redistribution of animals. *Nat. Clim. Change* **2**, 686–690 (2012).

33. Krashevska, V., Maraun, M. & Scheu, S. Micro- and Macroscale Changes in Density and Diversity of Testate Amoebae of Tropical Montane Rain Forests of Southern Ecuador. *Acta Protozool.* **49**, 17–28 (2010).

34. Homeier, J., Breckle, S.-W., Günter, S., Rollenbeck, R. T. & Leuschner, C. Tree Diversity, Forest Structure and Productivity along Altitudinal and Topographical Gradients in a Species-Rich Ecuadorian Montane Rain Forest: Ecuadorian Montane Forest Diversity and Structure. *Biotropica* **42**, 140–148 (2010).

35. Martínez-Camilo, R., González-Espinosa, M., Ramírez-Marcial, N., Cayuela, L. & Pérez-Farrera, M. Á. Tropical tree species diversity in a mountain system in southern Mexico: local and regional patterns and determinant factors. *Biotropica* **50**, 499–509 (2018).

36. Unger, M., Homeier, J. & Leuschner, C. Relationships among leaf area index, below-canopy light availability and tree diversity along a transect from tropical lowland to montane forests in NE Ecuador. *Trop. Ecol.* **54**, 33–45 (2013).

37. Peng, J. *et al.* Quality-assured long-term satellite-based leaf area index product. *Glob. Change Biol.* **23**, 5027–5028 (2017).

38. Vasconcelos, H. L., Vilhena, J. M. S., Facure, K. G., Albernaz, A. L. K. M. & Parr, K. Patterns of ant species diversity and turnover across 2000 km of Amazonian floodplain forest. *J. Biogeogr.* **37**, 432–440 (2010).

39. van Leeuwen, W. J. D. & Huete, A. R. Effects of standing litter on the biophysical interpretation of plant canopies with spectral indices. *Remote Sens. Environ.* **55**, 123–138 (1996).

40. Wallis, C. I. B. *et al.* Contrasting performance of Lidar and optical texture models in predicting avian diversity in a tropical mountain forest. *Remote Sens. Environ.* **174**, 223–232 (2016).

41. Wood, E. M., Pidgeon, A. M., Radeloff, V. C. & Keuler, N. S. Image texture as a remotely sensed measure of vegetation structure. *Remote Sens. Environ.* **121**, 516–526 (2012).

42. Homeier, J. & Leuschner, C. Factors controlling the productivity of tropical Andean forests: climate and soil are more important than tree diversity. *Biogeosciences* **18**, 1525–1541 (2021).

43. Krishna, M. P. & Mohan, M. Litter decomposition in forest ecosystems: a review. *Energy Ecol. Environ.* **2**, 236–249 (2017).

44. Staab, M., Schuldt, A., Assmann, T. & Klein, A. Tree diversity promotes predator but not omnivore ants in a subtropical Chinese forest. *Ecol. Entomol.* **39**, 637–647 (2014).
